# Dengue virus infection alters post-transcriptional modification of microRNAs in the mosquito vector *Aedes aegypti*

**DOI:** 10.1038/srep15968

**Published:** 2015-10-30

**Authors:** Kayvan Etebari, Solomon Osei-Amo, Simon Phillip Blomberg, Sassan Asgari

**Affiliations:** 1 Australian Infectious Disease Research Centre, School of Biological Sciences, The University of Queensland, Brisbane QLD 4072 Australia

## Abstract

Recent discoveries regarding the importance of isomiRs have increased our understanding of the regulatory complexities of the miRNAome. Observed changes in the miRNA profiles in mosquitoes infected with flaviviruses have implicated small RNAs in the interactions between viruses and their vectors. Here we analysed the isomiR profiles of both uninfected and infected *Aedes aegypti* mosquitoes with the major human pathogen dengue virus (DENV). We found that several specific isomiRs were significantly altered in their abundance patterns in response to DENV infection potentially affecting their target repertoire. Notable among these were isomiR variants which displayed arm-switching. We also demonstrate that modifications to the 3p end of miRNAs are vastly more prevalent than those at the 5p ends. We also observed that in only 45% of *Ae. aegypti* miRNAs the most abundant read matches the exact sequence reported in miRBase. Further, we found positive correlations between the number of mature miRNA reads, pre-miRNA length, GC content and secondary structure minimum free energy with the number of isomiRs. The findings presented here provide some evidence that isomiR production is not a random phenomenon and may be important in DENV replication in its vector.

MicroRNAs (miRNAs) are small non-coding RNA molecules, which have been demonstrated to have substantial impacts on gene regulation across a vast range of organisms through interaction with mRNAs^1^,^2^. Transcribed in the nucleus largely by RNA polymerase II, the primary miRNA (pri-miRNA) transcript is processed by the enzyme Drosha in association with an RNA binding protein, DGCR8 (or Pasha in insects) into a precursor (pre-miRNA) before being exported into the cytoplasm^3^,^4^. Once in the cytoplasm, pre-miRNA can then be further processed to generate the miRNA duplex consisting of a mature and a star strand miRNA^5^. It is either of these two strands (usually the mature strand) contained in the duplex, which are loaded into either argonaute (Ago) proteins Ago1 or Ago2 and directed to the mRNAs that they target^6^.

Recent findings have demonstrated that despite typically being annotated as a specific sequence, individual miRNAs often exist in a range of length and sequence variations termed isomiRs^7^,^8^,^9^,^10^,^11^. These variants or isomiRs were once thought to be sequencing errors but have since been demonstrated to be physiologically relevant and post-transcriptionally modified miRNA variants^12^,^13^. It has even been suggested that isomiRs may have affinities for different targets than their canonical miRNA counterparts^14^,^15^. Despite being largely more frequent at the 3′ end, these variations can occur at both ends of the miRNA sequence and can even be present in the form of nucleotide substitutions^10^,^16^,^17^,^18^. It is poorly understood how the production of isomiRs is regulated but there have been several proposed mechanisms as to the mode of their biogenesis, which has been demonstrated to be complex and even cell type specific^9^,^19^. Some of the variations observed in miRNA sequences might be the product of template variations brought about by the exonuclease activity of Drosha and Dicer^7^. When the substantial over-representation of 3′ modifications is taken into account, however, it is apparent that other factors contribute to the production of isomiRs. One suggested cause for this skewed distribution of isomiRs in favour of the 3′ variants is the stereochemistry of the complex formed between Ago proteins and miRNAs. Crystallographic studies indicate that the 5′ ends of miRNAs are shielded from exonucleolytic attack by the middle domain (MID) in Ago2^20^. Adenylation and uridylation by a host of nucleotidyl transferases have been experimentally determined to be predominantly responsible for 3′ variations in miRNA sequences. Furthermore, these modifications were found to be miRNA specific, implying some manner of regulatory control^9^. In plants, it is evident that these processes of uridylation and adenylation play antagonistic roles in regulating miRNA stability with un-methylated miRNAs being vulnerable to uridylation and subsequent degradation^21^,^22^. It is also relevant that for the most part, these nucleotidyl transferases catalyse the addition of nucleotides in a 5′ to 3′ directionally specific manner, making them more likely to add nucleotides to the 3′ end of a miRNA^7^,^23^. Modifications that result in “trimming” of the 3′ end of miRNA sequences are not accounted for by adenylation or uridylation. Studies in *Drosophila melanogaster* have suggested that trimming modifications to the 3′ end of miRNAs are enacted by the exoribonuclease “Nibbler”^24^,^25^. Also suggested in these studies, was the possibility of other Nibbler-independent mechanisms for 3′ trimming, as not all the miRNAs subject to 3′ trimming were affected by Nibbler knockouts.

Relatively little is known about the mechanisms responsible for 5′ miRNA modifications, however, in *Caenorhabditis elegans*, modifications to the 5′ end which destabilize miRNAs and contribute to their degradation *in vivo* have been shown to be enacted by the exoribonuclease XRN-2^10^. Despite their biogenesis being poorly understood, 5′ modifications to miRNAs are potentially crucially important given the tendency of particular base changes at the 5′ nucleotide to dictate which of the varying Ago proteins that a specific miRNA will bind to^26^,^27^.

MiRNAs may play a role in the ability of mosquitoes to act as vectors for widely damaging arboviruses such as dengue virus (DENV). For example, *Aedes aegypti* miRNAs have been shown to be modified in their abundance in the event of DENV infecting the mosquitoes^28^. Given our emerging understanding of the influence that variations in isomiR prevalence can have on gene regulation, a greater knowledge of the isomiR profile of mosquitoes responsible for the transmission of arboviruses is needed. In this study, we analysed the results of deep sequencing performed on the main DENV vector *Ae. aegypti* after DENV-infection as compared to uninfected mosquitoes^28^,^29^. We found that there were several specific miRNAs which showed alterations in their prevalence in response to DENV infection; however, this effect was not ubiquitous, with the overall pattern of isomiR expression not being significantly altered by DENV infection. These results indicate that at least for some miRNAs isomiR profiles could be dynamic with potential biological significance, which is as of yet undetermined.

## Results and Discussion

### IsomiRs and their prevalence in *Ae. aegypti*

We explored the sequence variation of mapped reads correlating with *Ae. aegypti* miRNAs in DENV-infected and uninfected mosquitoes. For this, we utilized publically available RNA-seq data from 2, 4, and 9-day DENV-infected and uninfected mosquitoes^28^. The absolute quantity and relative abundance of each class of isomiR (see Table 1 for definition of modifications) were measured in each library. It was clear from these data that the relative prevalence of each isomiR category was effectively unchanged with respect to DENV infection (Fig. S1, df:7 and *F* value = 0.07). The data also showed that the most commonly observed isomiR was the 3′ trim variant whilst the 3′ single nucleotide addition and the 5′ trim variants were comparatively rare (Fig. S1). The least common class of isomiR was the 5′ extension in which nucleotides matching the genomic precursor molecule sequence are added to the 5′ end of the miRNA sequence. Overall, isomiRs possessing modifications occurring at the 3′ end of miRNAs were vastly more abundant than those occurring at the 5′ end with a ratio of 25 to 1. These findings concur with the current literature^17^,^30^,^31^,^32^,^33^. It has been proposed that this enormous bias towards modifications to the 3′ end of miRNAs suggests that these variations are more than just artefacts generated by inaccuracies in data collection^15^. Sequence conservation at the all-important 5′ end is evidently a phenomenon whose existence is rooted in biological necessity as the 5′ terminal and seed (nt 2–8 from the 5′ end) regions of miRNAs have been implicated in their ability to affect their targets and even the possibility of affecting new targets^15^,^34^,^35^,^36^,^37^,^38^. It is therefore not surprising that these regions are more frequently conserved.

It appears likely that these sequence variations may indeed be generated by variations in the pre-miRNA sequences due to the activity of Drosha and Dicer, which are known to produce variations in transcript sequences^7^,^39^,^40^,^41^. Recently, it has been shown that about 70% of expressed miRNAs in human, mouse, *D. melanogaster* and *C. elegans* can produce 5′ modified isomiRs, and the mechanism responsible for this modification is perhaps well-conserved during evolution^35^.

### Alteration in isomiR production rate of specific miRNAs by DENV infection

Previously it has been reported that the isomiR expression can be different in response to various biological stimuli such as cancer and bacterial infection^9^,^13^,^42^,^43^,^44^. Recently, we also showed that modifications to miRNAs could occur as a result of *Wolbachia* colonisation in the dengue mosquito vector^45^. Here we found that DENV infection can also change the isomiR profile in *Ae. aegypti*.

**Figure 1:**
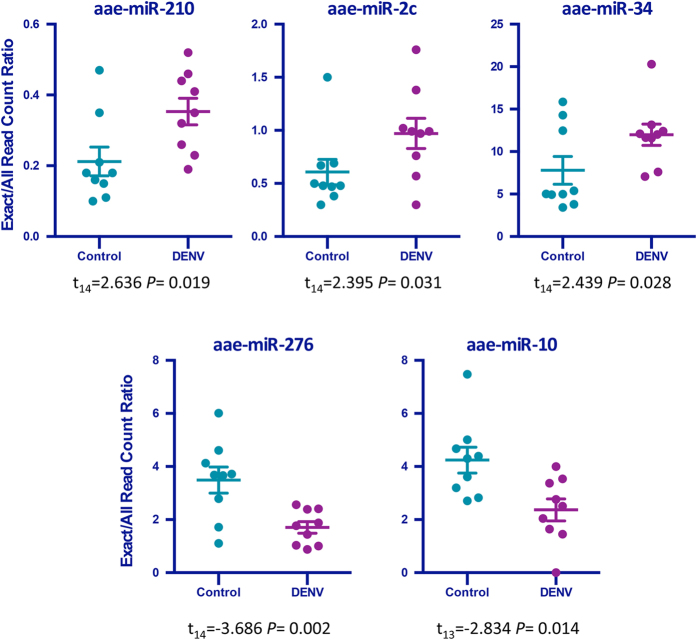
The canonical miRNA frequency modification in response to DENV infection for specific miRNAs. The ratio of the exact canonical miRNA sequence read count to its all different classes of isomiRs for those miRNAs whose ratio was significantly changed (*P* < 0.05).

To explore miRNA variation and post-transcriptional modifications, we calculated the exact/all read count ratio as an index for isomiR production rate for all known *Ae. aegypti* miRNAs. The reads which mapped exactly to mature miRNA sequences (extracted from miRBase version 20) were used to calculate the exact read counts for each miRNA. This read count significantly changed in five *Ae. aegypti* miRNAs upon DENV infection (Fig. 1). DENV infection significantly increased the isomiR production rate of miRNAs miR-2c, miR-210 and miR-34. Notable impact of DENV infection on exact/all read count ratio was also observed in miRNAs miR-276 and miR-10, whose read count was significantly reduced by DENV infection.

Taken together, these alterations to the mosquito isomiRome may have a collective net benefit or detriment to the mosquito’s ability to vector DENV. It is unclear what the potential biological significance of these modifications could be, but the fact that they are specific and seemingly varied amongst miRNAs does seem to suggest some evolved function. These findings are also congruent with many examples in the current literature, which also suggest targeted isomiR variation in response to differing biological contexts^44^,^45^,^46^. Given this variation with respect to the biological context of infection in this particular case, it would be interesting to test the isomiRome of other DENV vectors (mainly *Ae. albopictus*) and how they react to DENV. It is not necessarily a given that the isomiR profile changes we saw in these data, which were generated from RexD colony mosquitoes^28^, would be conserved among closely related strains. A study conducted by Loher *et al.* (2014) demonstrated that unique isomiR profiles exist in human lymphoblastoid cells, which differ between populations and even genders^47^. It is reasonable to conclude from this that isomiRomes can evolve rapidly and are potentially less conserved than miRNAomes.

### Specific classes of isomiRs modification due to DENV infection

**Figure 2:**
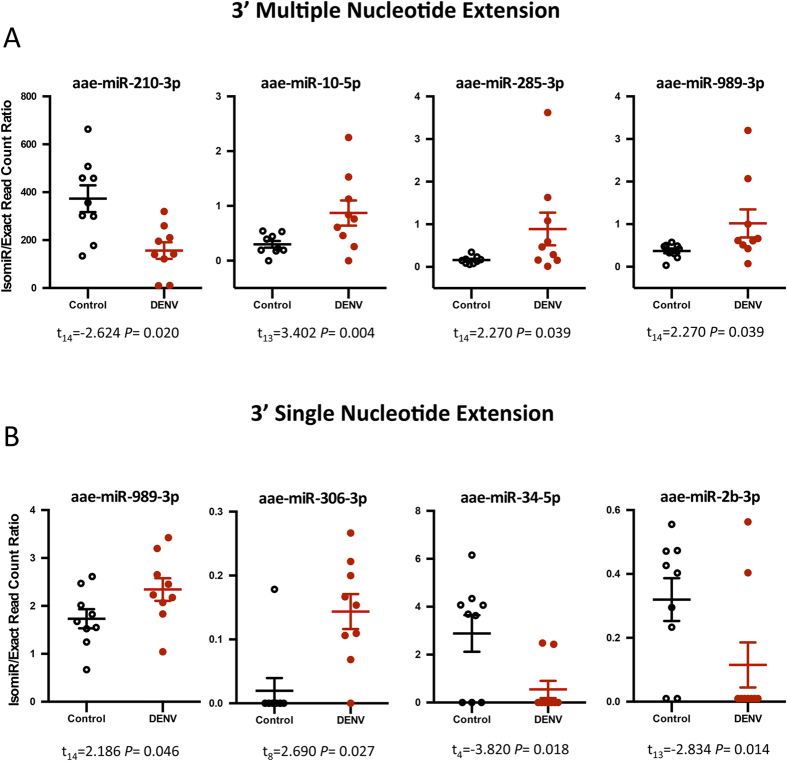
DENV infection altered the frequencies of 3′ end modified isomiRs in some miRNAs. The isomiR/exact read count ratio for (**A**) 3′ multiple nucleotides extension isomiRs, (**B**) 3′ single nucleotide extension isomiRs. The isomiR/exact read count ratio for these miRNAs was significantly altered due to DENV infection (*P* < 0.05).

**Figure 3:**
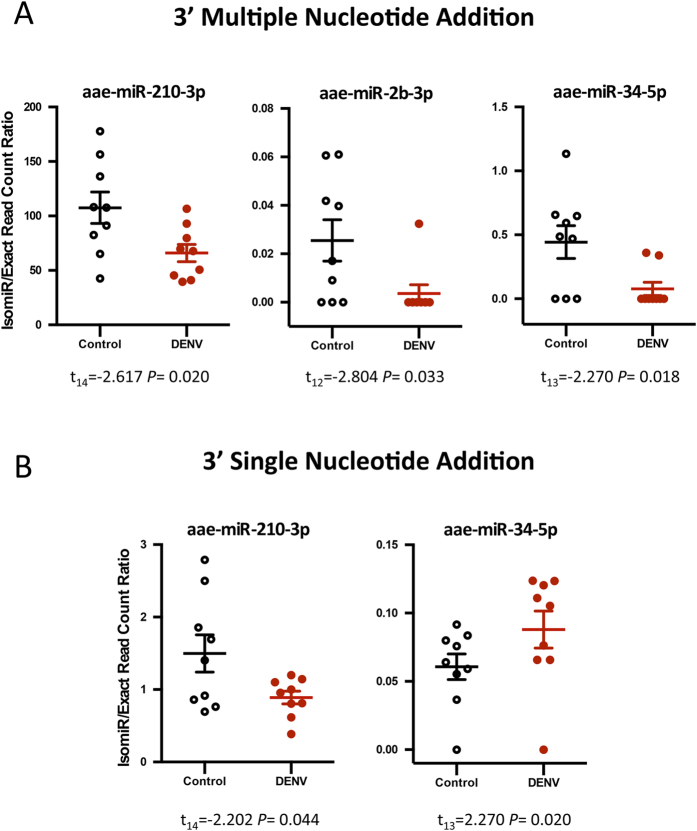
DENV infection modified the frequencies of 3′ nucleotides addition isomiRs in some miRNAs. The isomiR/exact read count ratio for (**A**) 3′ multiple nucleotides addition isomiRs, (**B**) 3′ single nucleotide addition isomiRs. The isomiR/exact read count ratio for these miRNAs was significantly altered due to DENV infection (*P* < 0.05).

**Figure 4:**
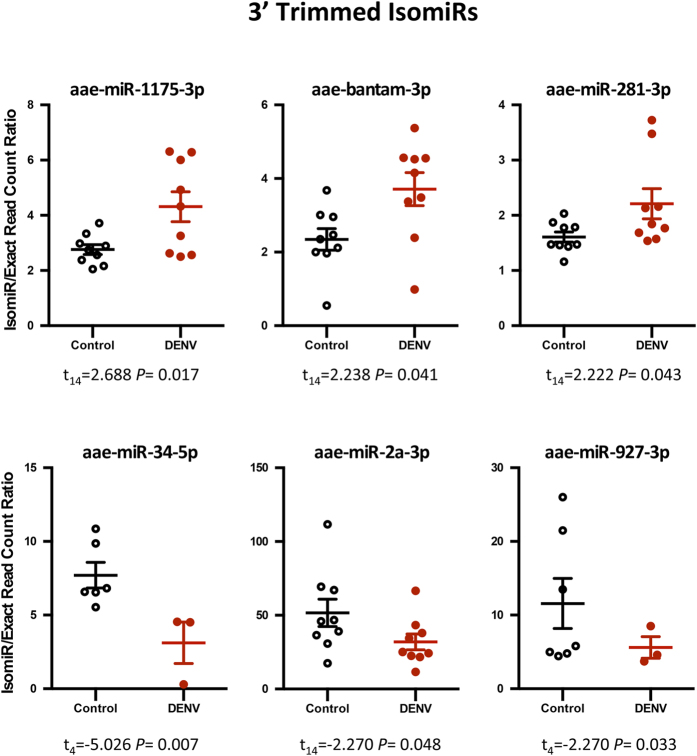
The change in the prevalence of 3′ trimmed isomiRs in response to DENV for specific miRNAs. The isomiR/exact read count ratio for these miRNAs was significantly altered due to DENV infection (*P* < 0.05).

Specific classes of isomiRs were observed to be considerably altered in their prevalence by DENV infection. The 3′ multiple nucleotide extension (3MNE) class of isomiRs displayed statistically significant induction in response to DENV infection in miR-285-3p, miR-989-3p and miR-10-5p (Fig. 2A). However 3MNE isomiR production rate considerably decreased for miR-210-3p in DENV infected samples. Significant changes in the read counts of the 3′ single nucleotide extension (3SNE) class of isomiRs with respect to DENV infection were also observed for miR-989-3p, miR-306-3p, miR-34-5p, and miR-2b-3p (Fig. 2B). Likewise, alteration in isomiR production ratio was detected for 3′ multiple nucleotide addition (3MNA) isomiRs in miR-210-3p, miR-2b-3p and miR-34-5p, which showed significant down-regulation in DENV infected mosquitoes (Fig. 3A). A similar effect was seen for 3′ single nucleotide addition (3SNA) for miR-210-3p, but an opposing effect for miR-34-5p (Fig. 3B). 3MNE and 3MNA isomiRs of miR-210-3p proved to be more highly abundant in DENV-infected mosquitoes in comparison with other miRNAs in the 3′ modification class of isomiRs. The 3′ trimmed (3′ Trim) class of isomiRs also varied in their abundance in response to infection; specifically miR-1175-3p, bantam-3p and miR-281-3p, showed significant induction, and miR34-5p, miR-2a-3p, and miR-927-3p significant reduction in infected mosquitoes (Fig. 4). The data also demonstrated that all 3′ isomiRs of miR-34-5p were significantly altered by DENV infection (Fig. S2). However, it is currently unclear as to why particular isomiR variants are more vulnerable to modifications in response to DENV in *Ae. aegypti*. It is interesting that miRNAs, which have significant increases in one particular isomiR variant in response to DENV, commonly have a variation in at least one other isomiR as well. It remains to be seen whether these alternate variants are equally biologically relevant or mere bi-products of miRNA biogenesis.

**Figure 5:**
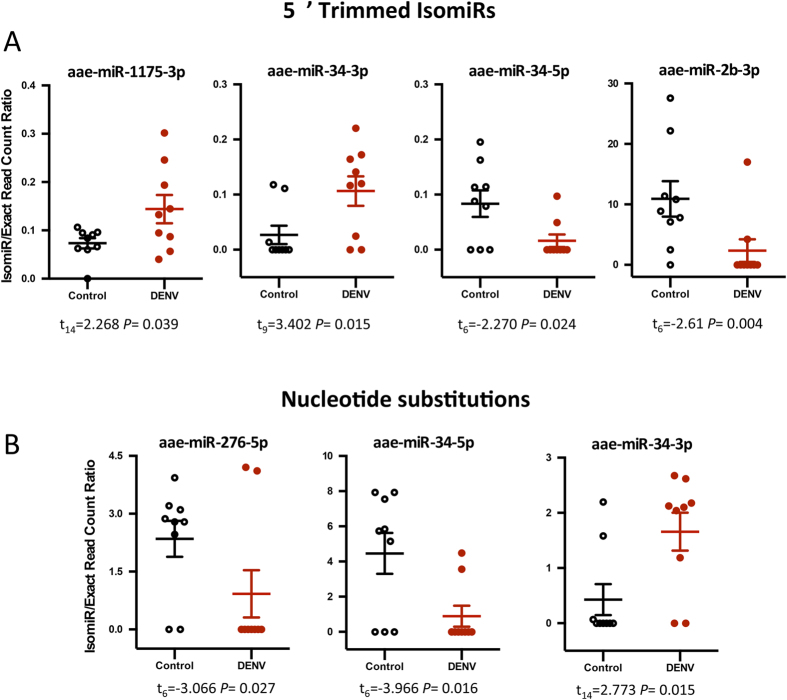
The change in the frequency of 5′ Trimmed and nucleotide substitutions isomiRs in response to DENV for specific miRNAs. The isomiR/exact read count ratio for these miRNAs was significantly (*P* < 0.05) altered due to DENV infection for (**A**) 5′ Trim and (**B**) Nucleotide substitutions.

DENV infection induced a significant increase in the abundance of 5′ Trim isomiRs for miR-34-3p and miR-1175-3p while 5′ Trim isomiRs were less abundant in miR-34-5p and miR-2b-3p in infected mosquitoes (Fig. 5A). However, we did not detect any significant modification due to DENV infection for other 5′ isomiRs.

The nucleotide substitution class of isomiRs appeared to be also affected by DENV (Fig. 5B). We observed significant variations to the isomiR/exact read counts of nucleotide substitution isomiRs in just three miRNAs, miR-276-5p, miR-34-5p and miR-34-3p (Fig. 5B). Given the significantly reduced rates of single nucleotide polymorphism (SNPs) observed in human miRNAs with respect to the overall genome, it has been suggested that there exist strong selective pressures, which act to conserve the sequence fidelity in miRNAs^48^,^49^. The data presented in this study provides evidence that the same selection pressures may act on *Ae. aegypti* mature miRNAs. We speculate that the establishment of DENV infection may cause changes to the activity levels of some enzymes involved in isomiR production and this might be a potential determinant of significant changes in isomiR frequencies of specific miRNAs after infection. For example, it is known that the enzyme responsible for 3′ trimming of miRNAs is the Nibbler protein^24^,^25^, however, there are currently no studies which describe the expression of Nibbler and other related enzymes with regards to infection in mosquitoes.

### Infection with DENV causes arm-switching in some miRNAs

**Figure 6:**
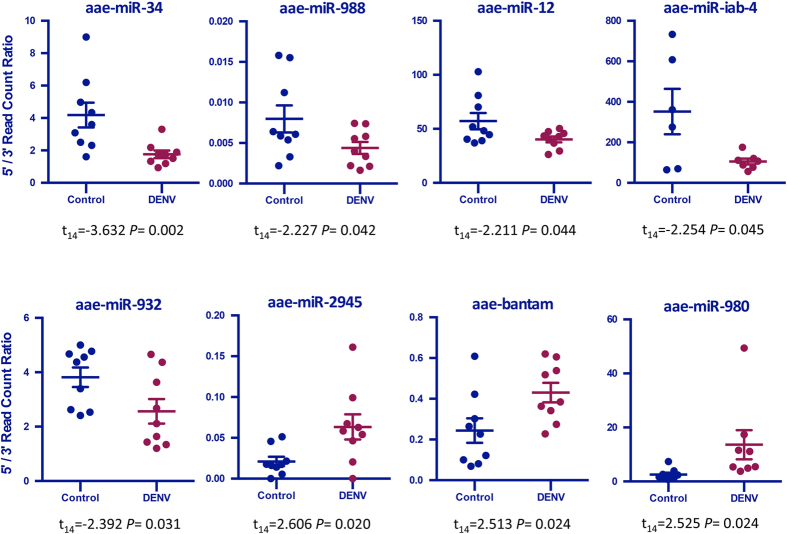
Ratio of 5p to 3p read counts in specific miRNAs. The series of graphs in this figure illustrate the ratio of miRNAs originating from the 5p arm of the hairpin structure to those originating from the 3p arm. The graphs show the ratios for those particular miRNAs whose change in ratio was statistically significant with respect to DENV infection (*P* < 0.05).

In general, an arm-switching event, change in the relative abundance of the 5p and 3p arms of a miRNA duplex, may indicate that a dominant product (5p or 3p) is selectively preferred. It is evident from the graphs in Fig. 6 that bantam’s ratio of 5p to 3p reads is considerably increased by DENV infection; this is also true for miR-980 and miR-2945. The 5p to 3p ratio was decreased by DENV infection for miR-34, miR-988, miR-12, miR-iab-4, and miR-932 (Fig. 6). The change in the predominant isomiR for these particular miRNAs likely represents a targeted and biologically significant modification of the mosquito host in response to infection; there are several published reports of such a phenomenon^32^,^42^,^50^,^51^. In these examples, incidences of infection or genetic disease resulted in the dominant isomiR of a miRNA to be shifted either to the opposite arm (3p) of the precursor or further up the 5p arm. As depicted in Figure S3, the most prevalent isomiR for miR-1000 in uninfected mosquitoes originates several nucleotides upstream from that, which is most prevalent in infected mosquitoes; it has shifted up the 5p arm of the precursor (Fig. S3A). In the case of miR-305, the most prevalent isomiR was found on the 5p arm in uninfected mosquitoes, however, for mosquitoes which were infected with DENV the most prevalent isomiR originated from the 3p arm, an arm-switching event in response to DENV infection (Fig. S3B). It is possible that these occurrences may also affect the target genes which the miRNAs interact with. This in turn could play an important role in mediating pathogenicity and infection.

Recently, Siddle *et al.* (2015) reported that in the context of mammalian host-pathogen interaction, isomiR profiles could be broadly modified by hosts in response to bacterial infection. This study indicated that there can be an overarching shift in both arm switching and sequence variability in response to pathogenic infection^44^, however, the changes were not always highly pronounced and often varied with respect to different pathogens. They also observed that particular miRNAs exhibited much more highly expressed isomiR variants differing to the canonical pathways than would be explained by the global trend. Furthermore, these effects were present in response to a particular bacterium. Such findings are consistent with those of this study, which indicate that specific miRNAs are more significantly affected with regards to their isomiR production than others in the presence of a symbiotic organism. A possible explanation for the lack of ubiquity of this phenomenon may be that DENV is not highly pathogenic to *Ae. aegypti*^52^ and therefore elicits only a moderate immunological response from the mosquito. This may also explain the relative temporal independence of our observations, which contrast to others. The lack of universal effect does however provide further credence to the notion that the particular isomiR variants, which are responsive to infection, are specifically targeted in some fashion. Our data therefore supports previous works which indicated that the relative isomiRs production levels are likely to be dynamic phenomena and might be miRNA-specific events^10^,^16^,^17^,^18^,^31^,^53^,^54^.

### IsomiR production power in different *Ae. aegypti* miRNAs

**Figure 7:**
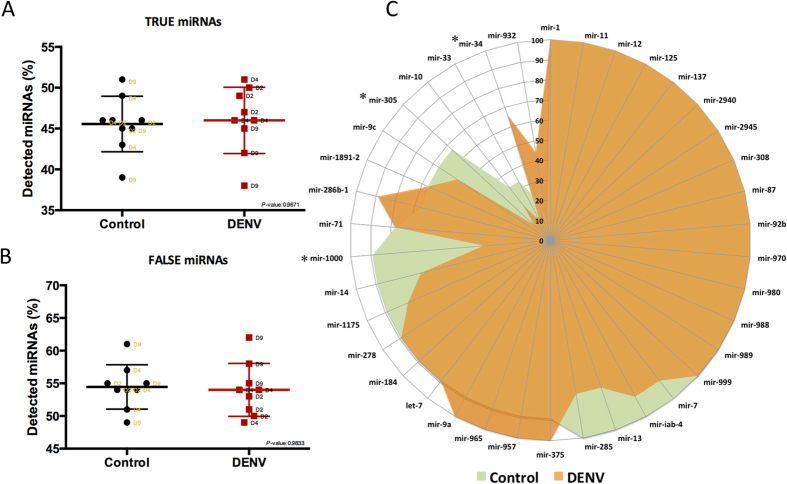
Less than half of *Ae. aegypti* canonical mature miRNAs were dominant isomiRs. (**A**) “True” miRNAs are those miRNAs for which the most highly expressed read was an exact match to the canonical (mature miRNA reported in miRBase) sequence. (**B**) For those miRNAs classified in the “False” group the most highly expressed read differed to that reported in miRBase. (**C**) The probability index for “True” miRNAs based on their canonical isomiR variation in all available libraries. MiRNAs which scored a probability index of 100% represent those miRNAs whose canonical sequences were the dominant isomiRs in all libraries.

We classified *Ae. aegypti* miRNAs into two classes based on the most highly expressed sequence as observed by read count. True miRNAs are those miRNAs for which the most highly expressed read was an exact match to the canonical sequence (mature miRNA reported in miRBase). For those miRNAs classified in the “False” group the most highly expressed read differed to that reported in miRBase (Fig. S4). The results indicated that for *Ae. aegypti*, the most abundant miRNA sequence matches the canonical miRNA in just 45% of cases, while 55% of *Ae. aegypti* miRNAs produce False miRNAs (Fig. 7A,B). It is also apparent that this overall trend is not altered by DENV infection. We examined the data from all available small RNA libraries and calculated a probability index for “True” miRNAs based on their isomiR variation in each library. In some miRNAs such as miR-1, miR-11, miR-12 and miR-999 (miRNAs with probability index of 100%) the canonical sequences were the dominant isomiR in all situations (Fig. 7C). Despite not having a global effect, in miRNAs miR-1000, miR-34, miR-305 and miR-10, the probability to be a “True” miRNA considerably changed due to DENV infection (Fig. 7C).

Our findings here are not surprising when they are considered in conjunction with the current literature. In a recent study of human dendritic cells subjected to bacterial infection, it was demonstrated that in only 50% of human miRNAs the canonical sequences were the dominant isomiR^44^. Also, in another small RNA deep sequencing analysis on human peripheral blood mononuclear cells, it was shown that the most highly expressed isomiR sequence of about 68% of miRNAs did not match with the reported miRNAs in the miRBase^31^. With the growing body of evidence indicating that a significant proportion of the annotated miRNA sequences are in fact isomiRs, which are not the dominant miRNA sequences, it is perhaps necessary to develop new criteria by which to denote the “True” sequence of miRNAs. In this case, the reference miRNA expression profile (previously reported in miRBase) may not deliver a precise picture of the miRNAome^7^,^31^. However, another possibility is that different biological conditions may affect the dominant miRNA sequence that is produced. Perhaps different tissues and environmental stimuli dictate which particular isomiR is most highly expressed making every miRNA sequence plastic in nature. If this is the case then the exact miRNA sequence is merely an arbitrary guide to identifying a group of miRNA isomiRs. There have also been some other reports of variation in the isomiR profiles of miRNAs across different tissues and cell types^16^,^17^,^30^. Given the knowledge that small variations in miRNA sequences can have significant impacts on their target affinities, this understanding is vital for the advancement of the field.

**Figure 8:**
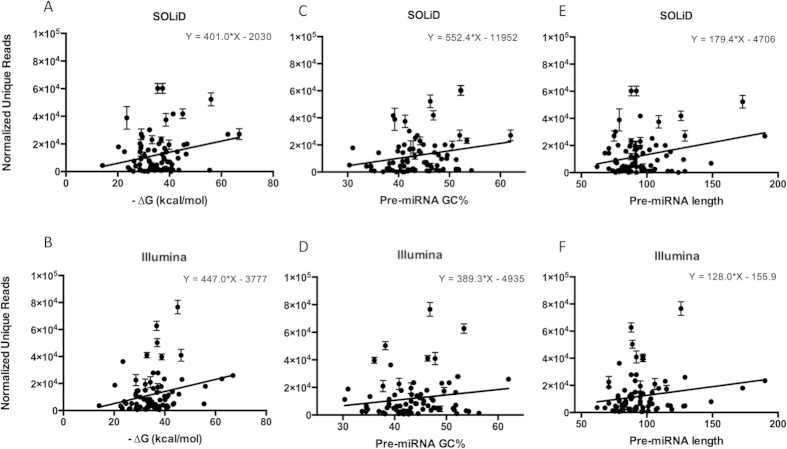
The impact of pre-miRNA structure on isomiR production power. There were significant correlations between the number of unique reads (isomiR enrichment) and structural parameters such as pre-miRNA secondary structure minimum free energy (**A**,**B**), GC content (**C**,**D**), and pre-miRNA length (**E**,**F**). These results are largely consistent between the two sequencing technologies.

To further identify the structural impact of pre-miRNA on isomiR production power for each miRNA, we plotted the normalized unique read data from all available libraries against pre-miRNA secondary structure minimum free energy (-ΔG), GC content, and pre-miRNA length. To evaluate the impact of deep sequencing platforms on isomiR production power, we analyzed two sets of data, one sequenced by SOLiD and one by the Illumina platform (Fig. 8). Our analysis showed that the number of isomiRs produced by each miRNA appears to have a positive correlation with the minimum free energy value of the pre-miRNA secondary structure (*P* value = 0.0312 and 0.0123 in SOLiD and Illumina, respectively). This data suggests that those miRNAs with lower minimum free energy, which thermodynamically are stronger, have greater isomiR production power (Fig. 8A,B). Similarly, the GC content of the pre-miRNA structure is associated with the number of isomiRs produced by the resultant miRNA (Fig. 8C,D). As the GC content increases, we observed on average, a higher number of variant isomiRs generated, but this correlation was not statistically significant in Illumina data (*P* value = 0.0221 and 0.1268 in SOLiD and Illumina, respectively). The length of pre-miRNA structure is also positively correlated with an increased number of observed isomiRs, as longer pre-miRNA lengths correspond to greater quantities of isomiRs (*P* value = 0.0187 and 0.0378 in SOLiD and Illumina, respectively). These results are largely consistent between the two sequencing technologies indicating that these phenomena are unlikely to be an artifact generated as a result of the sequencing method or data processing idiosyncrasies such as adapter trimming. These analyses also revealed that more highly expressed miRNAs produce more isomiRs or predominant unique reads (Fig. S5).

Taken together these findings seem to signify a relationship between the stability of a pre-miRNA structure and a prevalence of variant isomiRs. It is surprising, however, that the three indicators measured here (miRNA length, -ΔG, and GC content) as proxies for pre-miRNA stability, all seem to increase the number of variant isomiRs observed as they increase. Recently, a genetic analysis on let-7 secondary structure in 75 metazoan animal species showed that specific nucleotides were predominantly distributed in the 5′ and 3′ terminal loop ends, which may contribute to the relatively accurate cleavage that leads to a stable isomiR expression profile^55^. One enzyme that has been implicated in the production of isomiRs through the modification of pre-miRNA structures is the processing enzyme TRBP (trans-activation response (TAR) RNA binding protein)^56^. This enzyme can trigger the generation of unique miRNA sequencing reads by changing the site at which it processes the pre-miRNA^56^. Therefore, there exists a possibility that TRBP is somehow involved in the increase in isomiRs we observed in more stable pre-miRNA structures. If it was solely thermodynamic stability, which leads to the production of isomiRs, then we would likely see the opposite relationship. It is therefore necessary to consider the possible evolutionary significance of this relationship between pre-miRNA stability and isomiR production. It may be the case that pre-miRNA secondary structures, which are more stable, are more resistant to heritable alterations to their sequences and have thus accumulated more variations over time. Conversely, those pre-miRNA structures, which are not highly stable, may be quite vulnerable to loss of function with changes and such changes may therefore be less likely passed on.

### Fine-tuning gene expression by isomiRs

**Figure 9:**
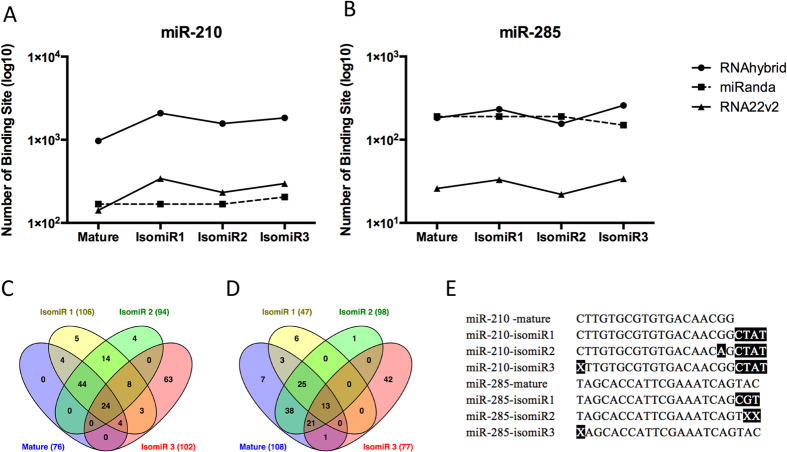
A Comparison of *in silico* target identification for different isomiRs of particular miRNAs. (**A**) The four sequences of miR-210 generated very similar target binding profiles between miRanda and RNA22 v2 but RNAhybrid generated far more predicted targets than either of them for all four sequences tested. (**B**) Both RNAhybrid and miRanda generated relatively similar target prediction profiles to each other across the four sequences of miR-285, whilst RNA22 v2 was disparate in that it generated far fewer potential binding sites across all aforementioned sequences. The Venn diagrams presented in (**C**,**D**) represent a subset of shared potential target genes for miR-210 and miR-285, respectively. The varying sequences used as isomiRs for the target identification pipeline are presented in (**E**). X, absence of a nucleotide in the position.

To compare the specificity of different isomiRs of two selected miRNAs (miR-210 and miR-285) on target regulation, we generated potential binding sites on all annotated *Ae. aegypti* 3′UTRs for each of the respective sequences and their isomiRs. Three commonly used target prediction programs RNAhybrid, miRanda and RNA22 v2 were used. We compared the data from the three programs. Selected genes with unique binding sites for each miRNA and its isomiRs, predicted by at least two programs, were selected as potential targets of high confidence. Overall, it was evident that the variations in sequences present in each isomiR can have potentially profound impacts on the number of predicted target sites generated by all programs (Fig. 9A,B).

In the case of miR-210, although a similar number of highly confident predicted target genes were predicted for all the three isomiRs, there were only a fraction of these (24 genes), which overlapped with each other as well as the mature miRNA sequence whose total potential targets were notably fewer (Fig. 9C). Similarly, the analysis on miR-285 predicted a very small proportion of potential target genes, which were conserved across all of the varying sequences (13 genes). In this case, however, the canonical miRNA sequence generated the highest number of potential targets (Fig. 9D), whilst miR-285 isomiR1, which contained a three-nucleotide extension at the 3′ end generated less than half of the potential binding sites in the combined model. Due to the importance of the seed region in all target prediction algorithms, isomiRs with 5′ end modification in both selected miRNAs (Fig. 9E) contained the highest number of unique potential target genes (63 and 42 genes for miR-210 and miR-285, respectively).

These findings suggest that isomiRs are not equal in their target gene regulation. Slight modifications to miRNA sequences may have significant impacts on their gene regulation activity. There have been few studies undertaken which explore the level of overlap in function between miRNAs and their isomiRs^50^,^57^,^58^,^59^. Baran-Gale *et al.* (2013) showed that just three potential target genes were shared among all predicted targets of three isomiRs for human miR-375^50^. Chan *et al.* (2013) also experimentally validated that miR-31 isomiRs display differential ability in repressing Dicer expression in MCF-7 breast cancer cells^57^. Other studies have suggested that different isomiR profiles can be expressed across different tissues^60^. Together, these findings add another layer of complexity to our ever-growing understanding of the interactions of the miRNA-mRNA network.

## Conclusion

There are an ever-growing number of reports, which illustrate the role that isomiRs play in the miRNA-mediated regulation of gene expression. These findings are increasingly suggesting that the production of isomiRs may be a regulated and imperative phenomenon in the normal functioning of organisms. Our findings have outlined the changes to the miRNAome with respect to different biological contexts in the major DENV vector *Ae*. *aegypti*. Although they showed no global correlations between changes in isomiR production with infection in mosquitoes, several individual miRNAs showed differential production of isomiRs in response to infection. When taken in conjunction, these results provide further evidence to the hypothesis that isomiR production is a regulated phenomenon and warrants further study with regards to the effects it may have on the ability of mosquito vectors to harbor viruses.

## Material and Methods

### Small RNA libraries

To track the post-transcriptional modifications of *Ae. aegypti* miRNAs, we used the publicly available small RNA libraries of DENV-2 infected and non-infected mosquitoes^28^,^29^. A total of 18 libraries with a study accession number of SRP026241 were downloaded from the National Center for Biotechnology Information Sequence Read archive. This data was generated by the SOLiD 3 sequencing platform from RNA samples of unexposed and exposed mosquitoes at three time points (day 2, 4 and 9 post-exposure, with three biological replicates). We also reanalyzed our previous small RNA deep sequencing data from DENV-2 infected and non-infected sheep blood-fed adult mosquitoes, which were sequenced by Illumina GA platforms. These data are accessible through NCBI’s Gene Expression Omnibus with GEO series accession number GSE59516.

### Computational pipeline for isomiR identification

CLC Genomic Workbench (version 7.0.4) was used to remove adapter sequences and reads with low quality scores from datasets. We applied the quality score of 0.05 as cut off for trimming. As described in CLC Genomic Workbench manual, the program uses the modified-Mott trimming algorithm for this purpose. The Phred quality scores (Q), defined as: *Q* *=* *−10log*_*10*_*(P)*, where P is the base-calling error probability, can then be used to calculate the error probabilities, which in turn can be used to set the limit for which bases should be trimmed. Hence, the first step in the trim process is to convert the quality score (Q) to an error probability: *P error* *=* *10^(Q/-10)*. Next, for every base a new value is calculated: *Limit – P error*. This value is negative for low quality bases, where the error probability is high. For every base, the Workbench calculates the running sum of this value. If the sum drops below zero, it is set to zero. The part of the sequence not trimmed is the region ending at the highest value of the running sum and starting at the last zero value before this highest score. Everything before and after this region was trimmed. A read was completely removed if the score never made it above zero.

To avoid any impurity in sequences, we also discarded the reads without 3′ adapters as well as those less than 16 nt in length after trimming. FASTQ/A Collapser of FASTX-toolkit was used to produce copy number based sequence lists. The tab separated files with the read sequences and their counts were used as input files for further analysis.

The automated workflow of miRanalyzer, a powerful web-based server for next generation sequencing analysis of miRNAs was used^61^. This software is designed based on a random forest classifier and implements a highly accurate machine-learning algorithm to provide an miRNA expression profile^61^. The miRanalyzer was selected for this study because of its popularity and accuracy in overall approach towards miRNA isoforms detection^62^. This tool uses the ultrafast short read aligner Bowtie (mapping seed alignment length: 17 nt) to align the reads to *Ae. aegypti* hairpin sequences of known miRNA data from miRBase version 20^63^. To detect more potential variations, we allowed three possible mismatches in the mapping criteria and all length variation and even non-template nucleotide additions collected as isomiRs, which are listed in Table 1.

We considered each unique mappable read to mature miRNA sequence as a potential miRNA isoform (isomiR). To observe the isomiR frequency for each type of defined isomiRs in all datasets, we calculated the ratios for isomiR/exact and also exact/all read count. The unique reads identical to the mature miRNA sequence reported in miRBase v20 were considered as the exact reads with which to compare the isomiR modification in each library.

To assess the impact of pre-miRNA structure on isomiR production rate, the pre-miRNA sequences were downloaded from miRBase and their stem loop secondary structures were predicted by a minimum free energy (mfe) approach in CLC Genomic Workbench^64^. The calculated mfe for each miRNA was plotted to normalized unique reads obtained from all SOLiD and Illumina small RNA libraries separately. We also checked the potential correlation between pre-miRNA GC content and their length, with isomiR production.

### *In silico* target identification for selected isomiRs

We selected the three most highly expressed isomiRs of two miRNAs (miR-210 and miR-285) for *in silico* target identification assays. All annotated 3′ UTR sequences of *Ae. aegypti* were downloaded from VectorBase^65^ and the potential binding sites for each miRNA were predicted by command line tools such as miRanda^66^, RNAhybrid^67^ and RNA22 v2^68^ algorithms using their default parameters. High confidence potential targets were defined as those containing a unique binding site for each miRNA in at least two algorithms, with a maximum of 10 nucleotides shifting.

### Statistical analysis

We fitted linear mixed-effects models (LMMs)^69^ to each isomiR dataset. For each miRNA we used Treatment (DENV-infected and non-infected) as a fixed factor with two levels and time points as a random effect (three time points with three biological replicates in total 9 for each miRNA). We used Index as the (log-transformed) response variable. We extracted the *p*-value for the Treatment effect (testing for a difference between infected and non-infected) for all miRNAs and Indices (Exact/all RC, isomiR/exact RC, 5′/3′ RC ratio). We did not adjust for multiple comparisons e.g. by adjusting *p*-values to control the false discovery rate^70^ or the family-wise error rate as these approaches were either too conservative or unreliable for small numbers of tests (<200)^71^,^72^. miRNAs were declared notable when *p* < 0.05 for any Index.

For the True/False data, we fitted a binomial generalized mixed-effects model (GLMM) with the same explanatory variables as above. The response variable was either one (True) or zero (False). We treated the *p*-values from each miRNA analysis as above. All analyses were performed in R^73^ using the packages nlme and lme4.

In order to test for differences in the relationship between pre-miRNA structural parameters (GC content, size and minimum free energy) and the number of unique reads for the two platforms (Illumina and SOLiD), we used a linear mixed-effects ANCOVA model, with (log-transformed) read number as a response variable, platform as a fixed effect and pre-miRNA parameters as a covariate. Read replicate and miRNAs were modelled as crossed random effects. Models were fitted using the lme4 package for R. Denominator degrees of freedom for F-tests of the fixed effects were estimated by Satterthwaite’s method using package lmerTest for R^74^. We checked for heteroscedasticity by plotting the residuals versus the fitted values. To check the assumption of normality of the residuals, we examined a Normal quantile-quantile plot. Both checks revealed no major departures from the model’s assumptions.

## Additional Information

**How to cite this article**: Etebari, K. *et al.* Dengue virus infection alters post-transcriptional modification of microRNAs in the mosquito vector *Aedes aegypti*. *Sci. Rep.* **5**, 15968; doi: 10.1038/srep15968 (2015).

## Supplementary Material

Supplementary Information

## Figures and Tables

**Table 1:** The isomiR types and their description^61^.

IsomiR Type	Code	Description
3′ single nucleotide extension	3SNE	The read is 1 nt longer than the reference mature sequence, but the nucleotide aligns with the hairpin sequence. The read starts at the same position in the hairpin than the reference mature sequence.
3′ multiple nucleotide extension	3MNE	The read is at least two nucleotides longer than the reference mature sequence, but the nucleotides align with the hairpin sequence.
3′ trimmed	3Trim	The read is shorter than the reference mature sequence but it starts at the same position in the hairpin.
5′ extension	5MNE	The read aligns to a position in the hairpin before the reference mature sequence but the last base of the read and the mature sequence map to the same position.
5′ trimmed	5Trim	The read aligns to a position in the hairpin after the reference mature sequence but the last base of the read and the mature sequence map to the same position.
Nucleotide substitutions	Subst	Sequence variation between the reference mature sequence and the read. Only those reads are considered that have the same length as the mature sequence and that start at the same position within the hairpin sequence.
3′ single nucleotide addition	3SNA	The read is 1 nt longer than the reference mature sequence, and the nucleotide does not align with the hairpin sequence. The read starts at the same position in the hairpin than the reference mature sequence.
3′ multiple nucleotide addition	3MNA	The read is at least two nucleotides longer than the reference mature sequence, and the nucleotides do not align with the hairpin sequence.
