# Supplementary material for: Dengue virus infection alters post-transcriptional modification of microRNAs in the mosquito vector Aedes aegypti
Source: Sci Rep. 2015 Oct 30;5:15968. doi: 10.1038/srep15968 (PMC4626843; doi:10.1038/srep15968)
Supplement: Supplementary Information [file srep15968-s1.pdf]

Title: Dengue virus infection alters post-transcriptional modification of microRNAs in the mosquito vector *Aedes aegypti*

Kayvan Etebari, Solomon Osei-Amo, Simon Phillip Blomberg and Sassan Asgari

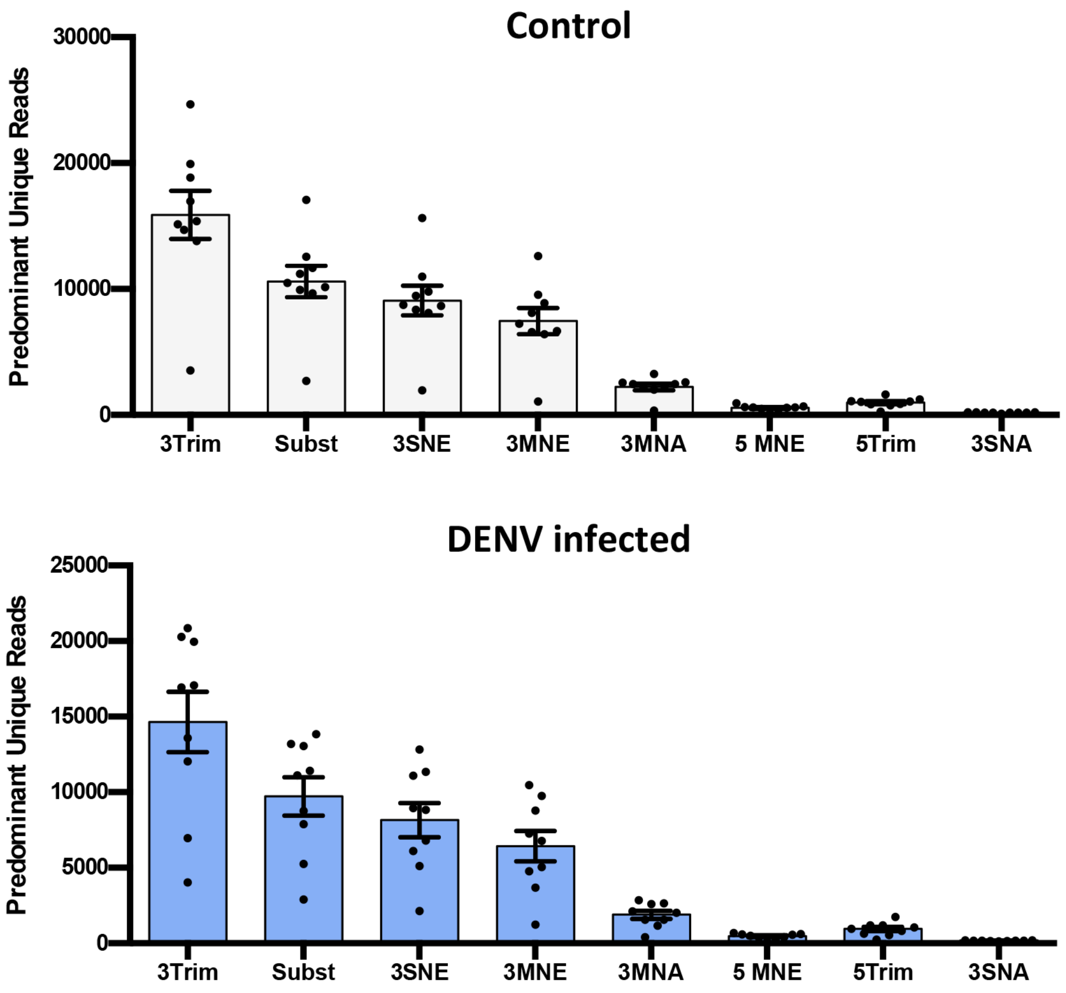

Figure S1. Different isomiR prevalence across DENV infected and non infected. Comparison of the proportion of unique reads for each type of isomiR for infected and non-infected mosquitoes (averaged across three time point and three biological replicates). Unique sequences with 3' modification were more abundant than 5' trimmed or extended sequences in all *Ae. aegypti* small RNA libraries. There were no considerable differences among all libraries (DenDf=124, P value= 0.07). 3SNE: 3' single nucleotide extension, 3MNE: 3' multiple nucleotide extension, 3Trim: 3' trimmed, 5MNE: 5' extension, 5Trim: 5' trimmed, Subst: nucleotide substitutions, 3SNA: 3' single nucleotide addition, 3MNA: 3' multiple nucleotide addition.

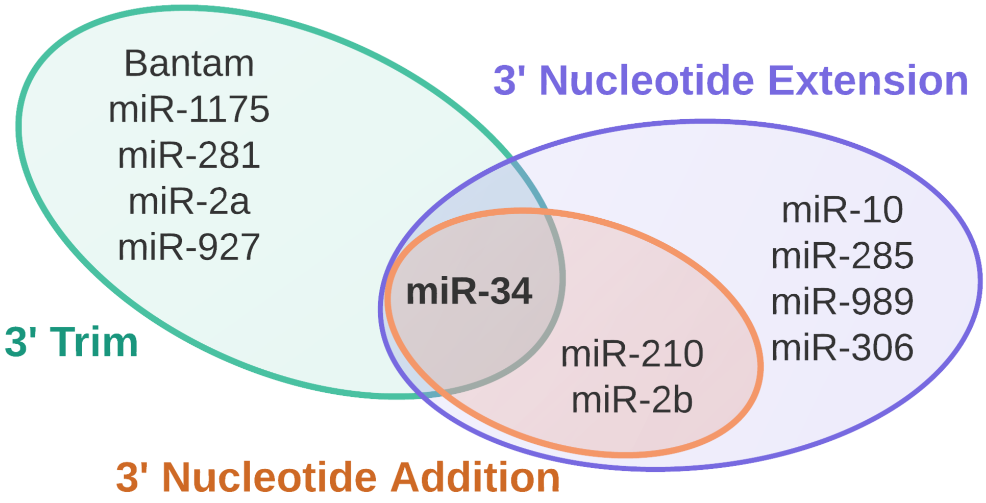

Figure S2. Venn diagram represent overlapped miRNAs with significant altered 3' isomiRs due to DENV infection. miR-34 is the only miRNA whose all 3' end modified ismiRs significantly altered by DENV infection (*P*<0.05).

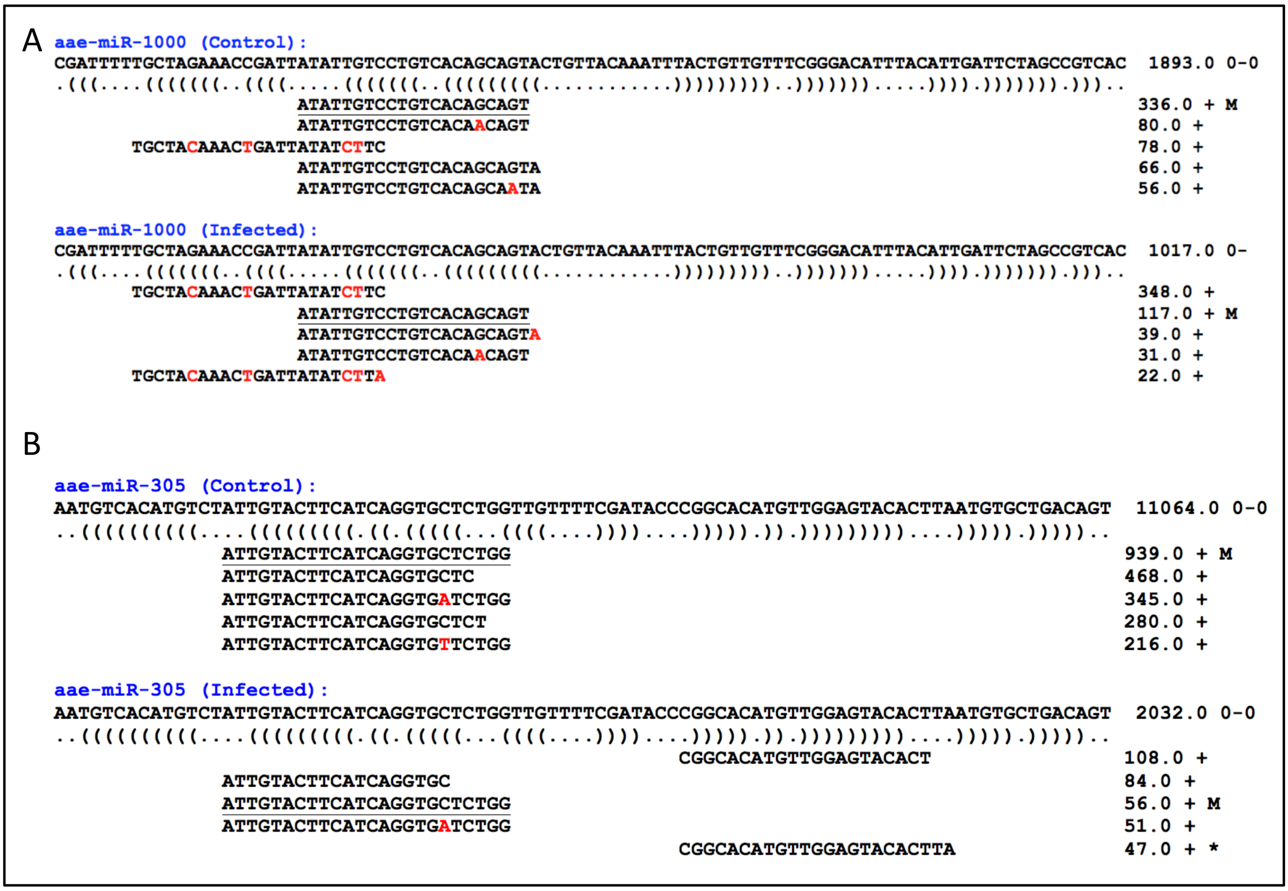

Figure S3. Schematic representation of two examples of arm-switching event. This figure displays schematically the area from which the most abundant isomiR read originates from within its precursor for both miRNAs (A) aae-mir-1000 and (B) aae-miR-305 in uninfected and DENV-infected treatments. Shifting of mature miRNA occurred in miR-1000 after infection and an arm-switching event happened in miR-305. The highly expressed miRNA was detected in 5p arm of the control group, which switched to 3p arm in infected mosquitoes (B).

A **Control day 2**

```
AUGACUCUUGUGCGUGUGACAACGGCUAUUG  23908.0 0-0
.))).)))))))))).))).)).))))).....
      CUUGUGCGUGUGACAACGGCUAU    5527.0 +
      CUUGUGCGUGUGACAACGACUAU    1910.0 +
      CUUGUGCGUGUGACAACAGCUAU    1481.0 +
       UUGUGCGUGUGACAACGGCUAU    994.0 +
       UUGUGCGUGUGACAACGACUAU    476.0 +
      CUUGUGCGUGUGACAACGGCUAUU   448.0 +
      CUUGUGCGUGUGACAACGGCAAU    432.0 +
      CUUGUGCGUGUGACAACGGCU      379.0 +
      CUUGUGCGUGUGACAACGGCUAC    275.0 +
      CUUGUGCGUGUGACAACAACUAU    266.0 +
      CUUGUGCGUGUGACAACGGCUAUA   250.0 +
       UUGUGCGUGUGACAACAGCUAU    249.0 +
      CUUGUGCGUGUGACAACGGCUAAU   234.0 +
      CUUGUGCGUGUGACAACGACU      221.0 +
      CUUGUGCGUGUGACAACGGCUAA    162.0 +
      CUUGUGCGUGUGACAACGCCUAU    160.0 +
      CUUGUGCGUGUGACAACGGAU      156.0 +
      CUUGUGCGUGUGACAACGGCUAUC   150.0 +
      CUUGUGCGUGUGACAACAGCU      121.0 +
      CUUGUGCGUGUGACAACGGCUAUG   119.0 +
      CUUGUGCGUGUGACAACGACUAUA   117.0 +
      CUUGUGCGUGUGACAACGGCAUAU   115.0 +
      CUUGUGCGUGUGACAACGGCCAU    110.0 +
      CUUGUGCGUGUGACAACGGAUAU    110.0 +
      CUUGUGCGUGUGACAACAGCAAU    104.0 +
      CUUGUGCGUGUGACAACGUCUAU    104.0 +
      CUUGUGCGUGUGACAACGGCC      101.0 +
      CUUGUGCGUGUGACAACGGACU     100.0 +
      CUUGUGCGUGUGACAACGACAAU    94.0 +
      CUUGUGCGUGUGACAACAGCUA     91.0 +
      CUUGUGCGUGUGACAACGACUAC    79.0 +
      CUUGUGCGUGUGACAACGGUAU     79.0 +
      CUUGUGCGUGUGACAACGCUAU     79.0 +
      CUUGUGCGUGUGACAACGGCAUA    78.0 +
      CUUGUGCGUGUGACAAUGGCUA     76.0 +
      CUUGUGCGUGUGACAACGGUUAU    75.0 +
      CUUGUGCGUGUGACAACGGCUACU   75.0 +
      CUUGUGCGUGUGACAACGUAGCG    75.0 +
      CUUGUGCGUGUGACAACCGCUAU    74.0 +
      CUUGUGCGUGUGACAACAGCUAC    73.0 +
       UUGUGCGUGUGACAACGGCAAU    71.0 +
      CUUGUGCGUGUGACAACGACUAUU   70.0 +
       UUGUGCGUGUGACAACAACUAU    70.0 +
      CUUGUGCGUGUGACAACGGCUAG    66.0 +
      CUUGUGCGUGUGACAACGACUA     66.0 +
      CUUGUGCGUGUGACAACGCGAUA    63.0 +
      CUUGUGCGUGUGACAACAGCCAU    62.0 +
      CUUGUGCGUGUGACAACGGCUAGU   62.0 +
       UUGUGCGUGUGACAACGGCUAC    58.0 +
      CUUGUGCGUGUGACAACGACUAA    57.0 +
      CUUGUGCGUGUGACAACGGCUA     57.0 +
                                 56.0 +
      CUUGUGCGUGUGACAACGGCAAAU   55.0 +
      CUUGUGCGUGUGACAACGGCUAUAU  54.0 +
       UUGUGCGUGUGACAACGGCUAUA   53.0 +
      CUUGUGCGUGUGACAACGACCAU    52.0 +
       UUGUGCGUGUGACAACGGCUAUU   52.0 +
      CUUGUGCGUGUGACAACGGC       50.0 +
      CUUGUGCGUGUGACAAAGGCUA     50.0 +
      CUUGUGCGUGUGACAACGACUAUG   49.0 +
      CUUGUGCGUGUGACAACGG        49.0 + M
```

Canonical miR-210

B **Infected day 2**

```
ACUCUUGUGCGUGUGACAACGGCUAUUG  14493.0 0-
).))))))))).))).)).)))))....
   CUUGUGCGUGUGACAACGGCUAU    3267.0 +
   CUUGUGCGUGUGACAACGACUAU    907.0 +
   CUUGUGCGUGUGACAACAGCUAU    735.0 +
   CUUGUGCGUGUGACAACGGCU      468.0 +
    UUGUGCGUGUGACAACGGCUAU    416.0 +
   CUUGUGCGUGUGACAACGGCUAUU   254.0 +
   CUUGUGCGUGUGACAACGGCUAC    226.0 +
   CUUGUGCGUGUGACAACAACUAU    217.0 +
   CUUGUGCGUGUGACAACGGCAAU    176.0 +
    UUGUGCGUGUGACAACGACUAU    168.0 +
   CUUGUGCGUGUGACAACGGCUAAU   168.0 +
   CUUGUGCGUGUGACAACGACU      139.0 +
   CUUGUGCGUGUGACAACGGCUAUA   123.0 +
    UUGUGCGUGUGACAACAGCUAU    120.0 +
   CUUGUGCGUGUGACAACAGCU      105.0 +
   CUUGUGCGUGUGACAACGGCC      92.0 +
   CUUGUGCGUGUGACAACGGCCAU    89.0 +
   CUUGUGCGUGUGACAACGGCAUAU   85.0 +
   CUUGUGCGUGUGACAACGGAU      81.0 +
   CUUGUGCGUGUGACAACGGCUAUC   81.0 +
   CUUGUGCGUGUGACAACGGCUAA    80.0 +
   CUUGUGCGUGUGACAACGGCUA     78.0 +
   CUUGUGCGUGUGACAACGGCUAUG   78.0 +
   CUUGUGCGUGUGACAACGCCUAU    77.0 +
   CUUGUGCGUGUGACAACGCUAU     72.0 +
   CUUGUGCGUGUGACAACGGC       70.0 +
   CUUGUGCGUGUGACAACCGCUAU    70.0 +
   CUUGUGCGUGUGACAACGGACU     67.0 +
   CUUGUGCGUGUGACAACGGAUAU    61.0 +
   CUUGUGCGUGUGACAACGGCUAGU   60.0 +
   CUUGUGCGUGUGACAACGACUAUA   58.0 +
   CUUGUGCGUGUGACAACGGCUACU   57.0 +
   CUUGUGCGUGUGACAACGGUAU     55.0 +
   CUUGUGCGUGUGACAACGGGCU     55.0 +
   CUUGUGCGUGUGACAACGACUAC    54.0 +
   CUUGUGCGUGUGACAACGACUAUG   50.0 +
   CUUGUGCGUGUGACAACAGCUA     49.0 +
   CUUGUGCGUGUGACAACGACUAUU   48.0 +
   CUUGUGCGUGUGACAACAGCAAU    47.0 +
   CUUGUGCGUGUGACAACGGCUAUAU  46.0 +
   CUUGUGCGUGUGACAACGGCAAAU   46.0 +
   CUUGUGCGUGUGACAACAGCUAC    45.0 +
   CUUGUGCGUGUGACAACAGCCAU    45.0 +
   CUUGUGCGUGUGACAACGGCUAG    45.0 +
   CUUGUGCGUGUGACAACGUCUAU    45.0 +
   CUUGUGCGUGUGACAACGG        45.0 + M
```

Figure S4. Changes in the number of unique reads produced by miR-210 in infected and control groups. Highlighted in yellow are all those sequences which matched the exact miR-210 as annotated in miRBase. (A) IsomiRs expressed more highly than the canonical miR-210 in the control group. (B) The DENV-infected group exhibited a reduced number of unique reads.

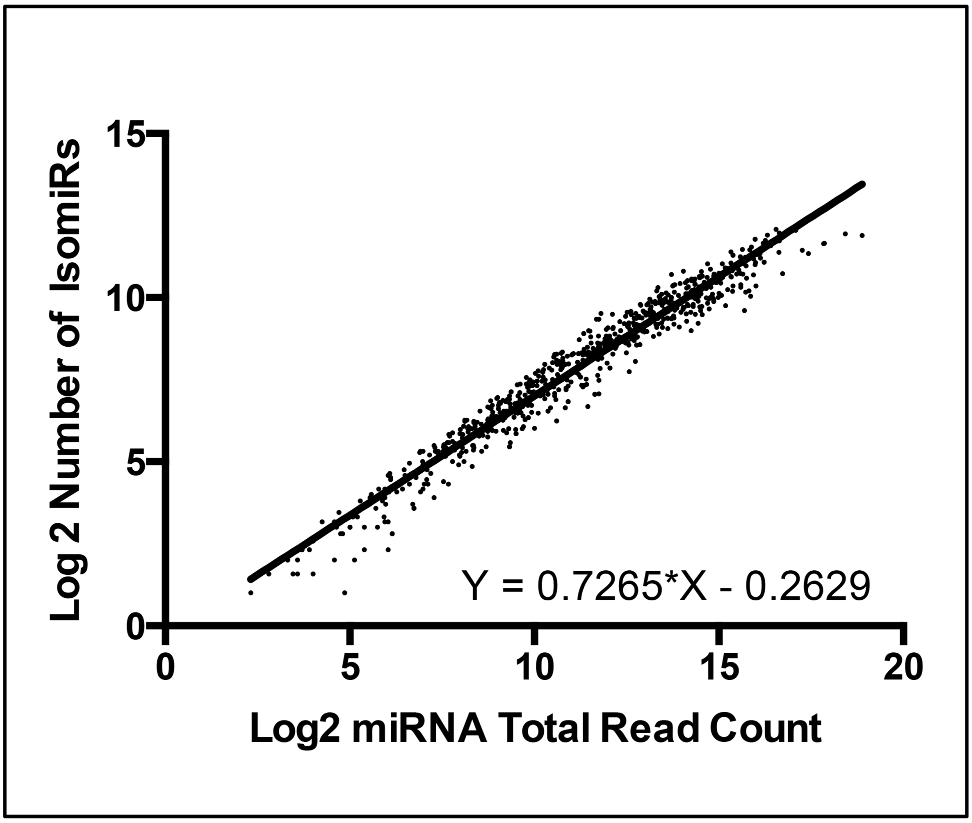

Figure S5. The linear relationship between miRNA expression and isomiR production. The graph depicts the relationship between the read count of miRNAs and the total number of unique reads or isomiRs produced by those miRNAs. For more highly expressed miRNAs, a greater number of unique reads were generated.
